# Impact of blocking layers based on TiO_2_ and ZnO prepared via direct current reactive magnetron sputtering on DSSC solar cells

**DOI:** 10.1038/s41598-024-61512-6

**Published:** 2024-05-09

**Authors:** Maciej Sibiński, Paulina Sawicka-Chudy, Grzegorz Wisz, Paweł Gnida, Ewa Schab-Balcerzak, Andrzej Wal, Rostyslav Yavorskyi, Marian Cholewa

**Affiliations:** 1 Department of Material and Environmental Technology, Tallinn University of Technology, Ehitajate tee 5, 19086 Tallinn, Estonia; 2 Department of Semiconductor and Optoelectronic Devices, Łódź University of Technology, Al. Politechniki 10, 93-590 Łódź, Poland; 3 Institute of Materials Engineering, College of Natural Sciences, University of Rzeszów, Pigonia 1, 35-310 Rzeszów, Poland; 4 Centre of Polymer and Carbon Materials, Polish Academy of Sciences, 34 M. Curie-Sklodowska Str., 41-819 Zabrze, Poland; 5 Institute of Chemistry, Faculty of Mathematics, Physics and Chemistry, University of Silesia, Szkolna 9, 40-007 Katowice, Poland; 6 Institute of Physics, College of Natural Sciences, University of Rzeszów, Pigonia 1, 35-310 Rzeszów, Poland; 7 Department of Physics and Chemistry of Solid State, Vasyl Stefanyk Precarpation National University, T. Shevchenko Str. 57, Ivano-Frankivsk, 76-018 Ukraine

**Keywords:** Engineering, Materials science, Optics and photonics

## Abstract

The optimization of dye-sensitized solar cells (DSSCs) technology towards suppressing charge recombination between the contact and the electron transport layer is a key factor in achieving high conversion efficiency and the successful commercialization of this type of product. An important aspect of the DSSC structure is the front blocking layer (BL): optimizing this component may increase the efficiency of photoelectron transfer from the dye to the semiconductor by reduction charge recombination at the TiO_2_/electrolyte and FTO/electrolyte interfaces. In this paper, a series of blocking layer variants, based on TiO_2_ and ZnO:TiO_2_, were obtained using the reactive magnetron sputtering method. Material composition, structure and layer thickness were referred to each process parameters. Complete DSSCs with structure FTO/BL/m-TiO_2_@N719/ EL-HSE/Pt/FTO were obtained on such bases. In the final results, verification of opto-electrical parameters of these cells were tested and used for the conclusions on the optimal blocking layer composition. As the conclusion, application of blocking layer consists of neat TiO_2_ resulted in improvement of device efficiency. It should be noted that for TiO_2_:ZnO/Cu_x_O and TiO_2_/Cu_x_O cells, higher efficiencies were also achieved when pure TiO_2_ was used as window layer. Additionally it was proven that the admixture of ZnO phase inspires V_oc_ and FF growth, but is overall unfavorable compared to pristine TiO_2_ blocking layer and the reference cell, according to the final cell efficiency.

## Introduction

In modern times, the demand for electricity is steadily increasing. Consequently, the search for more efficient sources of energy has intensified. Simultaneously, the challenges posed by climate change impose additional constraints on energy generation devices. These devices should be environmentally friendly, i.e. emit as little carbon dioxide as possible both during their production and operation. Moreover, their manufacturing and subsequent disposal should not lead to further intergenerational (toxic) waste. This is an opportunity for the better use and integration of renewable energy sources, such as solar, wind and geothermal energy. Access to such energy sources, while maintaining low costs for their conversion into useful energy, is of great importance for the development of a more conscientious and forward-thinking global community.

A vast amount of solar energy reaches the Earth’s surface, with an estimated power 1.75 × 10^7^ W^1^. However, its utilization is constrained by climatic factors (weather) and the absence of continuous availability. The search for effective solutions for its conversion to electricity remains a subject of interest for researchers. One response to these challenges involves the ongoing improvement of the efficiency of traditional silicon-based photovoltaic cells and the enhancement of the efficiency of the third generation of cells. This includes cells in which dyes participate in the conversion of photon energy into electrical current.

Research in the field of photoelectrochemistry was carried out in the twentieth century, yet the proposal for the construction of dye-sensitized solar cells (DSSCs) was introduced in the work of Gratzel and O’Regan^2^. Since then, researchers have made great efforts to increase the efficiency of such cells and to improve the stability of their work (see review papers^3–7^).

### Construction and parameters of dye-sensitized solar cells

Dye-sensitized solar cells, in comparison to traditional silicon cells, offer cost advantages stemming from the use of inexpensive materials, relatively simple production processes, and flexibility. They are also less toxic and perform well under a variety of lighting conditions^8^, including low-light conditions (dawn, dusk and cloudy weather or even using diffused light)^3,4^. Additionally, these structures often possess bifacial characteristics, or semi-transparency, and various coloration which extend the application field greatly, including in important building integrated photovoltaics (BIPV) sectors.

Some disadvantages of DSSCs include instability caused by electrolyte leakage and the degradation of platinum, which is used to achieve good conductivity. An additional negative feature is the use of synthetic dyes that contain toxic compounds^3^. Various solutions have been proposed to mitigate these issues. Instead of expensive conductive FTO or ITO, layers based on graphene could be used. Additionally, solid electrolytes can replace liquid electrolytes^3,9^. Theoretical studies on the losses occurring in dye cells, and ways of reducing them, are also conducted^10^.

**Figure 1:**
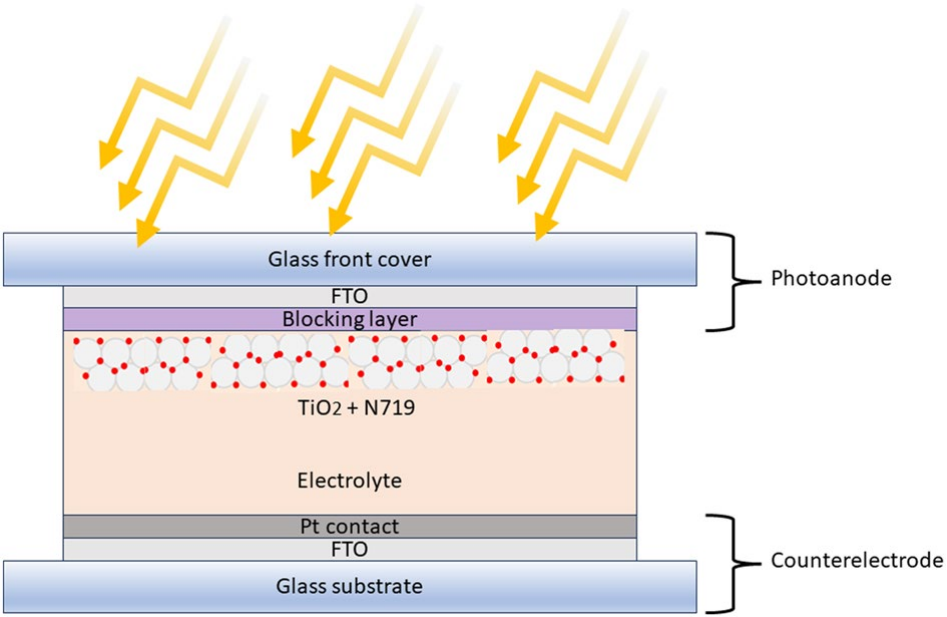
Scheme of DSSCs with the structure investigated in the paper (glass/FTO/(blocking layer)/m-TiO_2_@N719/EL-HSE/Pt/FTO/glass).

A standard dye cell consists of four components: a photoanode made of a mesoporous oxide layer (usually titanium oxide) connected to a glass conductive layer; a dye sensitizer; an electrolyte containing a redox pair; and a counter electrode^3^. The electrolyte is typically an iodide/triiodide redox couplet dissolved in a nonaqueous solvent. It ensures the stabilization of the photosensitizer by supplying electrons to it, and in turn it is regenerated by the counter electrode, which is connected by a circuit with the photocathode^1^. Figure 1 presents typical construction of DSSC.

These components of dye-sensitized solar cells are undergoing intensive study to find solutions that will ensure their practical use. The design of the photoanode should provide the largest surface area to which the dye molecules attach, while ensuring the effective transfer of excited electrons to the conductive layer (usually platinum contact on FTO glass) through the semiconductor forming the electrode discussed^5^. The material used for the construction of the photoelectrode is usually titanium oxide TiO_2_, although other oxides such as ZnO, SnO_2_, Nb_2_O_5_ or Zn_2_SnO_4_ are also used^11^. In the case of ZnO, to prevent low cell efficiency, surface modification of this material is used with the application of noble metal nanoparticles^11^.

An additional requirement is placed on the dyes attached to the photoelectrode: appropriate relationships between the energy levels of electrons in the dye and the semiconductor forming the photoanode must be ensured for the transport of light-induced electrons of the dye to the conduction bands of the semiconductor. Due to low cost and availability, compelling candidates are cells based on natural dyes, which are also environmentally friendly without causing pollution^3,9,12^. Hence, research is being carried out to replace dyes produced with the use of rare metal complexes by organic dyes. The production of such metal-free organic dyes is based on the use of structures of the D-D-π-A type^8^. To improve the efficiency of dye cells, the electrolyte used should also be considered. Due to the limited temperature ranges of operation of liquid electrolytes, new types of electrolytes, e.g. solid or gel electrolytes are being developed, which eliminate the disadvantages of evaporation and leakage^13^.

### Impact of the blocking layer on the performance of the DSSC

Despite intensive research, the efficiency of dye photocells remains around 14%^14^. The dependencies of efficiency on electrical parameters indicate that to increase the conversion efficiency of light into electricity, three parameters should be elevated: the open-circuit voltage (V_oc_); the short-circuit current density (J_sc_); and the fill factor (FF)^3^. One way to achieve such changes is to increase the efficiency of the photoelectron transfer from the dye to the semiconductor. Further, charge recombination occurs at the TiO_2_/electrolyte and FTO/electrolyte interfaces, causing a decrease in the short-circuit current^8,13,15,16^. A solution to reduce the recombination process is to create a blocking layer (BL) between the conductive glass (e.g. FTO) and the mesoporous semiconductor. This layer should protect the FTO from contact with the electrolyte, and be thin enough to avoid a significant increase in circuit resistance^15^. Metal oxides such as ZnO, TiO_2_, Al_2_O_3_, Nb_2_O_5_, MgO, ZrO, Ga_2_O_3_ and SiO_2_ are commonly used to produce blocking layers^8^. There are several techniques for obtaining blocking layers: atomic layer deposition^17^; spray pyrolysis; electrochemical deposition^18^; magnetron sputtering^15^; and sol–gel methods^19^.

Research has been conducted to investigate the impact of blocking layers on the operating parameters of dye cells. In the work of Xia et al.^20^ it was shown that the introduction of a thin layer of Nb_2_O_5_ between fluorine-doped tin oxide FTO and nanocrystalline TiO_2_ significantly improved V_oc_ and the fill factor (FF), which caused an increase in cell efficiency (PCE). Scanning Electron Microscopy (SEM) and cyclic voltammetry (CV) studies have shown that the layer acts as an electronic lock rather than a morphological lock at the FTO/TiO_2_ interface. The authors explain the increase in efficiency by the layer’s participation in extinguishing charge recombination processes. Similar results regarding the impact of the blocking layer on V_oc_ and FF parameters were obtained by Kabir et al.^9^. In this case, the blocking layer was TiCl_4_ placed on the FTO/TiO_2_ interface, which reduced the recombination of charge carriers and introduced a rapid transport path. To compare the effect of the blocking layer, tests were carried out on a cell based on a natural dye (betacyanin) with and without the BL. It was observed that the layer increases the short circuit current by approximately 24% and improves efficiency by approximately 28%. Additionally, stability studies were carried out on both versions of cells, indicating that the introduction of the blocking layer does not alter the nature of the time dependence of cell efficiency.

A significant increase in the electron lifetime measured by electrochemical impedance spectroscopy was also observed for such a layer. Blocking layers also increase the efficiency of electrochemical water oxidation, as shown by Zhang et al.^21^. Samples with different blocking layer thicknesses were produced using the atomic layer deposition method between mesoporous TiO_2_ and FTO. The researchers showed that such layers hinder the recombination of electron–hole pairs. Table 1 summarizes the photovoltaic parameters of the cells containing the N719 dye, which was also used in the preparation of this manuscript.

**Table 1:** Photovoltaic performance of DSSC cells with N719 dye containing blocking layers.

Material of blocking layer	Thickness [nm]	V_oc_ [mV]	J_sc_ [mA cm^−2^]	FF	PCE [%]	References
None	–	610	3.95	0.53	1.27	^22^
ZnO	5	610	5.86	0.49	1.75	
ZnO	7	620	6.09	0.53	2.02	
ZnO	12	640	7.13	0.56	2.57	
ZnO	15	630	6.66	0.56	2.37	
None	–	880	8.10	0.71	5.10	^22^
SnO_2_	60	870	8.80	0.72	5.60	
SnO_2_	120	830	13.30	0.69	6.10	
None	–	770	14.50	0.66	7.37	
SnO_2_	60	790	14.51	0.67	7.92	
SnO_2_	120	820	15.84	0.65	8.38	
None	–	732	8.12	0.70	4.15	^24^
TiO_2_	13	732	8.72	0.71	4.51	
TiO_2_	25	764	9.42	0.72	5.16	
TiO_2_	50	750	8.92	0.70	4.71	
None	–	710	16.36	0.63	7.37	^25^
TiO_2_	115	730	16.88	0.65	8.02	
TiO_2_	131	770	16.34	0.67	8.42	
TiO_2_	182	770	17.23	0.65	8.55	
TiO_2_	321	750	17.16	0.65	8.37	
None	–	676	4.87	0.58	1.90	^26^
TiO_2_	60	719	8.16	0.58	3.39	
TiO_2_	78	714	5.74	0.63	2.58	
None	–	690	3.60	0.58	1.45	^27^
TiO_2_	150	650	5.14	0.49	4.63	
TiO_2_	300	690	5.80	0.52	2.07	
TiO_2_	450	670	6.10	0.43	1.75	
None	–	676	4.87	0.58	1.90	^26^
TiO_2_/Nb_2_O_5_	60	719	8.16	0.58	3.39	
TiO_2_/Nb_2_O_5_	78	714	5.74	0.63	2.58	

According to the data in Table 1, it can be seen that, in most cases, the use of blocking layers resulted in an increase in the short-circuit current density (with the exception of BL TiO_2_ with a thickness of 131 nm) and the efficiency of the photovoltaic cells tested. However, it is worth noting the dependence of the recorded photovoltaic parameters on the thickness of the compact layer. Taking into account the research results from the cited works, it is not possible to select a single optimum blocking layer thickness, bearing in mind that all these cells contained the commercial dye N719. Hence, the conclusion is that the selection of the compact layer thickness is very important and depends on the specific device preparation conditions. Most likely, it cannot be unified for all solar cells of similar design. It is noteworthy that the range of blocking layer thicknesses is very large, (from 5 to 450 nm), and furthermore, in most groups (with the exception of the results in the paper^23^) the best values rather are not recorded for the thinnest or thickest layers, confirming that the relationship between thickness and recorded parameters is not linear, as in the case of mesoporous layer thicknesses^28^.

In our previous works, we attempted to increase the efficiency of TiO_2_/CuO cells by replacing the TiO_2_ layer with a TiO_2_:ZnO layer^29^. The including of the ZnO wurtzite phase under appropriate conditions induces columnar growth of the TiO_2_ layer and improves its transport properties. The use of different targets leads to the generation of vapor streams with different characteristics and composition, which causes different growth processes and layer formation. In the case of TiO_2_:ZnO/CuO cells, the highest efficiency was achieved using a Ti-ZnO target. For a more comprehensive exploration of the influence of blocking layer morphology and opto-electric parameters on dye solar cell behavior, we conducted an investigation reported in this article. In this article, these TiO_2_:ZnO layers and a reference TiO_2_ blocking layers were obtained using the reactive magnetron sputtering method. The complete DSSCs were fabricated and characterized. A comprehensive study of the layer production method was also delivered. Various deposition variants of blocking layer production were proposed and tested according to their application in selected DSSCs Specific layer analyses were amended by the measurement of the full set of constructed cell parameters. Determining the correlation between performance and blocking layer parameters depending on the type of target used requires additional experiments and will be the subject of analysis in future work.

### Sputtering process of blocking layer

As is well known, in sputtering methods thin films are produced via the condensation of molecular streams of substance onto the surface of a solid body (substrate). The physical properties of thin films depend on the atomic structure and their perfection^30–33^. They, in turn, are determined by the kinetics of the film formation process. The process of forming thin films is complex, involving adsorption, nucleation of a new phase, growth, clustering, cluster coalescence, and so on^34^. We will consider the temperature range in which the thermal density fluctuations in the film are negligibly small compared to the density of the substance on the sample where the film forms.

Adsorption precedes the formation of a film during condensation from a gaseous environment. An atom approaching the substrate enters the range of van der Waals attractive forces, which at a closer distance from the surface are replaced by repulsive forces. At a certain distance from the substrate, denoted as “r_a_”, the atom has a minimum potential energy, E_a_, which is the atom’s adsorption energy. This corresponds to the adsorbed state of the atom – adatom. The value of E_a_ represents the binding energy of the atom to the substrate and is equal to the work required to detach the atom from the surface and convert it to a free state^35^.

Since atom re-evaporation is possible, its lifetime near the surface is limited and is determined by the expression τ_α_ = ν^−1^exp(E_a_/k_B_T)^36^, where k_B_ is the Boltzmann constant, T is the substrate temperature, ν is the frequency of atom jumps during surface migration, which is of the same order of magnitude as the frequency of thermal vibrations of an atom in a crystal lattice node (10^12^–10^13^ Hz). During the time τ_a_, the adsorbed atom migrates on the surface, forming a two-dimensional pair with a temperature equal to the substrate temperature^37^.

In the initial stage of deposition, the density of the atoms increases rapidly, reaching an equilibrium value n_e_ = Nτ_α_, where N is the intensity of atoms hitting the surface. If n_ν_ is the surface density of atoms in a saturated two-dimensional pair, then condensate formation occurs when n_e_ > n_ν_. The critical condensation temperature T_c_, above which the formation of a stable condensate on the substrate does not occur, is determined by the condition n_ν_ = Nτ_a_ = Nν^−1^ exp(E_a_/k_B_T_c_)^37^.

Under supercritical conditions, migrating atoms on the surface collide with each other. According to the Frankel theory, form stable centers consisting of two bonded atoms. The desorption energy of such a center increases with the energy of the chemical bond between the atoms. As a result, they remain on the surface longer and manage to attach to the next atom. This is how a cluster of atoms is formed, representing the nucleus of a new phase, which is bound to the substrate for a longer period and tends to grow further by absorbing other migrating atoms on the surface^37^. As a result of the growth of the nucleus of the new phase, a stable nanoscale region is formed, which is represented by a nanocluster^38^.

**Figure 2:**
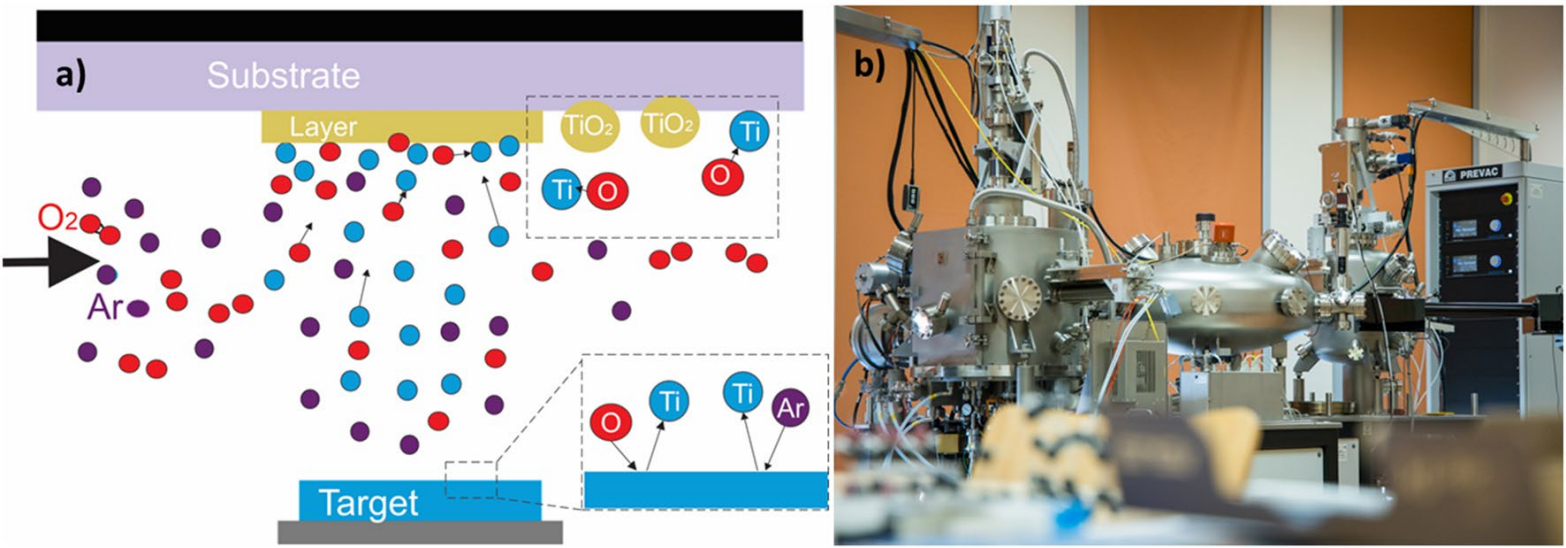
TiO_2_ blocking layer deposition process scheme (**a**) and laboratory setup used by the authors (**b**).

The further formation of a thin film in the initial stage is determined by two main mechanisms: the capture of atoms by a nanocluster and the coalescence of nanoclusters themselves. The influx of atoms into the nanocluster occurs in two ways: capturing surface-migrating adatoms via diffusion, and capturing atoms from the two-dimensional pair formed during deposition^39,40^. The blocking layer reactive direct current magnetron sputtering deposition process scheme for Ti target and laboratory equipment used by authors are presented in Fig. 2.

It should be noted that the formation of connected atomic complexes on the surface also leads to the formation of surface vacancies. This results in a vacancy-atom complex, which is so stable that it can be considered as a subcritical nucleus of a new phase. As a result, the subsurface vacancies become direct condensation centers. In the models proposed in this work, we will consider the surface as ideal, where only the subcritical nuclei of the new phase serve as condensation centers. The process flow of blocking layer forming can be divided into stages taking place in the near-surface stable area and the transition area, as shown in the Table 2.

**Table 2:** Blocking layer growth process flow.

Transition balance zone	Adsorption
	Surface diffusion
	Adatom condensation
Film formation/stable region	New phase nucleation
	Stable nanocrystal formation
	Crystallite growth

## Experimental section

### Growth of TiO_2_ZnO thin film blocking layers

Three TiO_2_:ZnO and one TiO_2_ thin film blocking layers were deposited by the DC (direct current) reactive magnetron sputtering method using ZnOTiO_2_, Ti-Zn, Ti-ZnO and Ti targets. The ZnO:TiO_2,_ Ti (99.995%) targets and ZnO pellet were provided by the Kurt J. Lesker Company. TiO_2_:ZnO layers were selected which, when used in TiO_2_:ZnO/CuO cells, allowed to obtain their best photovoltaic parameters^29^. A different type of target was used in each process, which required the use of a different set of deposition process parameters.

The preparation process for the Ti-Zn and Ti-ZnO targets were as follows: in a titanium Ti target with a diameter of 1 inch and a thickness of 4 mm, a hole was drilled with a diameter of 3.2 and 9.6 mm. Subsequently, the Zn (for Ti-Zn target) and ZnO (for Ti-ZnO target) pellet with the same thickness was placed, and finally the targets were compressed.

TiO_2_ZnO thin film blocking layers were deposited on the commercial transparent glass-coated Fluorine doped thin oxide (FTO) plates with an area of 1.0 ± 0.1 cm^2^, and a resistance of R < 7 Ω/sq Base pressure was below 10^−6^ mbar before deposition began. The substrate temperature during the deposition of the thin films was kept at 300 °C, and 200 °C (for sample ZnOTiO_2_). O_2_/Ar mixtures with a ratio of 3.5:0.5; 3:1; 2,5:1, were used as an active gas in the sputtering process.

Details of the sputtering conditions and the approximate value of the thickness of the TiO_2_:ZnO and TiO_2_ blocking layers are listed in Table 3.

**Table 3:** Growth parameters of the TiO_2_ZnO thin films blocking layers.

Parameter	Sample codes			
	ZnOTiO_2_	Ti-Zn	Ti-ZnO	Ti
Target	ZnOTiO_2_	Ti-Zn	Ti-ZnO	Ti
Time [min]	30	30	30	20
Power [W]	100	100	100	100
Pressure [mbar]	9.89 × 10^−3^	9.66 × 10^−3^	1.17 × 10^−2^	1.05 × 10^−2^
Distance between the source and substrate [mm]	58	58	58	58
Oxygen flow rate [cm^3^/s]	3.5	3.5	3	2.5
Argon flow rate [cm^3^/s]	0.5	0.5	1	1
Substrate temperature [°C]	200	300	300	300
Thickness [nm]	51	67	329	35

### DSSC preparation

Fluorine doped tin oxide coated glass slides (FTOs, 7 Ω/sq), Ru (II) (2,2′-bipyridyl-4,4′-dicarboxylic-acid) (2,2′-bipyridyl-4,4′-ditetrabutylammonium-carboxylate) (NCS)_2_ (N719), tert-Butanol, acetonitrile, electrolyte (EL-HSE) were purchased from Sigma Aldrich. 18NR-T Titania Paste, surfactant and isopropanol (IPA) were purchased from Greatcell Solar Materials, Hellmanex III, Hellma Analytics and POCH, respectively.

**Figure 3:**
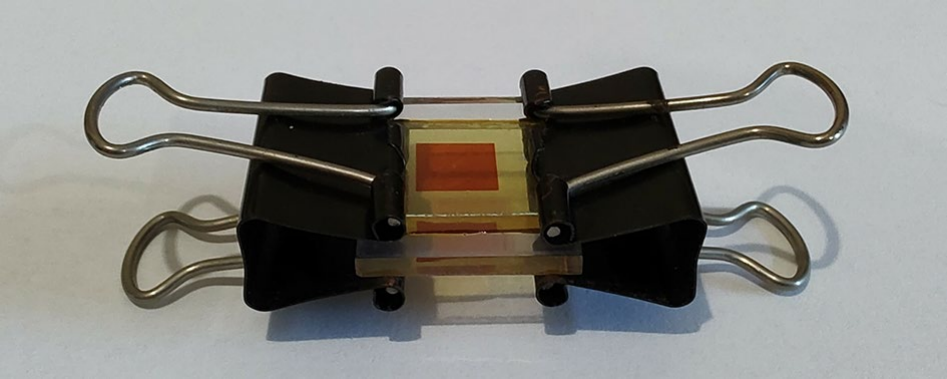
Real photo of semi-transparent, bifacial DSSC device obtained in the experiments.

The FTO substrates covered by blocking layers were dried and TiO_2_ layers approximately 10 µm thick were screen-printed onto them. To achieve this thickness, three layers of TiO_2_ paste were applied, each time drying the substrate at 125 °C. Finally, substrates with applied TiO_2_ layers were annealed at 500 °C in air for 30 min. To prepare the photoanodes, the fabricated oxide substrates heated to 80 °C were immersed in solutions of the respective dyes at a concentration of 3 × 10^−4^ M for 24 h. The mixture of ACN and *tert*-butyl alcohol (1:1) was used to prepare the dye solution. After this time, the substrates with adsorbed dye molecules were removed from the solution and rinsed with ethanol. The fabricated photoanodes were employed to assemble clamped devices, seen below, with the following structure: glass FTO/TiO_2_@N719/EL-HSE/Pt/FTO/glass. The counter-electrode was nano platinum applied on FTO glass. The commercial liquid electrolyte consists of I^−^/I_3_^−^ redox couple was placed between electrodes. The photo of this mechanism is shown in Fig. 3.

### Photoanode and DSSCs characterization

The UV–Vis absorptions spectra of TiO_2_ with adsorber N719 were recorded using a V-570 UV–Vis–NIR Spectrophotometer (Jasco Inc.). The morphology of the surface of photoanodes in nanoscale was characterized by atomic force microscopy (AFM) using a TopoMetrix Explorer device, operating in contact mode, in air, in constant force regime. The devices were tested using a PV Solutions Solar Simulator and a Keithley 2400 (Tektronix, Inc., Beaverton, OR, USA) under AM 1.5 G illumination (1000 W m^−2^).

## Results and discussion

### Optical properties

**Figure 4:**
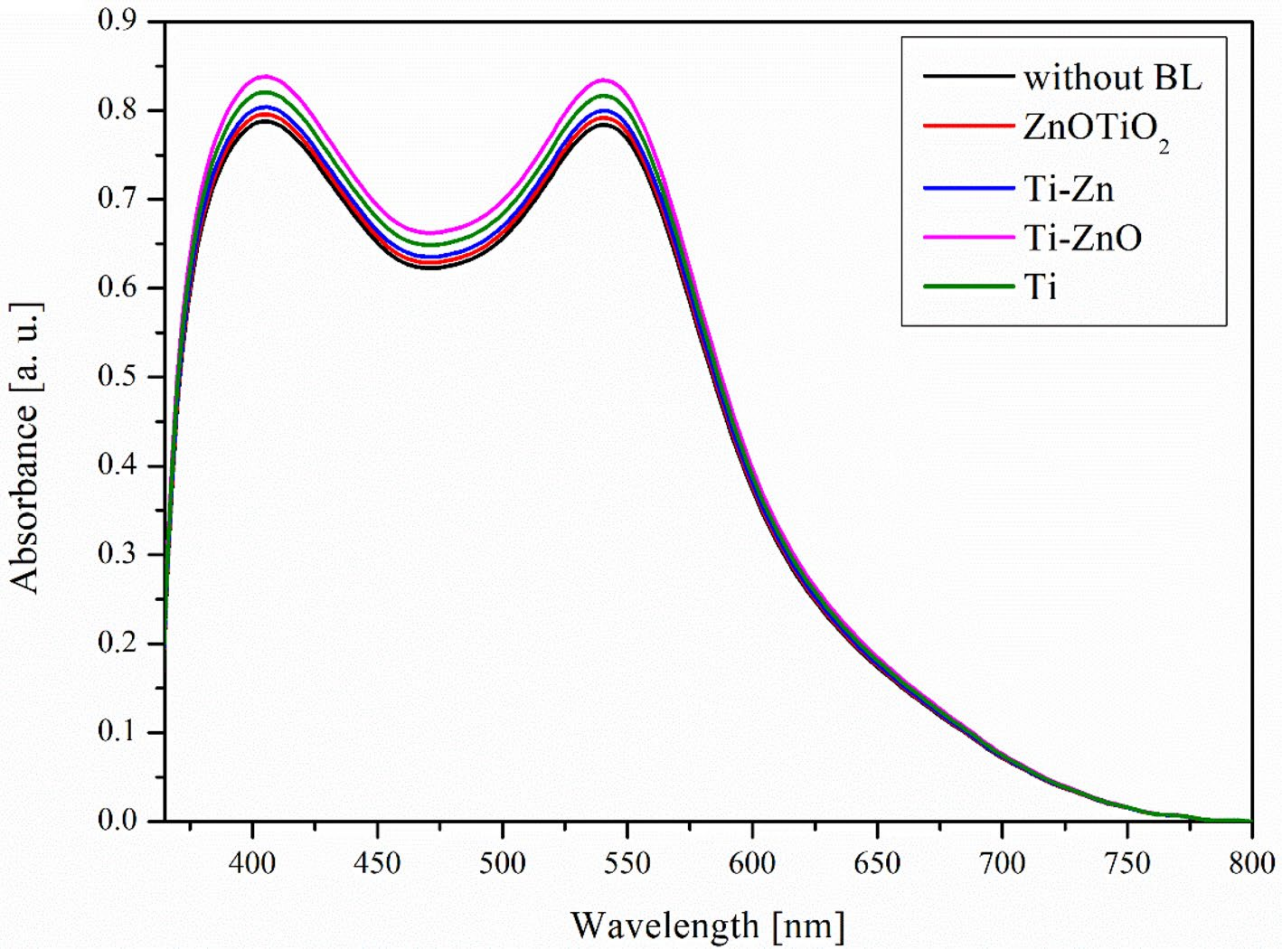
UV–Vis absorbance spectra of prepared photoanodes with anchored dye N719 and various BL.

Based on the UV–Vis absorption studies carried out, it was found that the use of blocking layers obtained by magnetron sputtering containing different compositions (TiO_2_/ZnO) did not affect the significant differences in absorbance and the range of absorbed light. The recorded spectra are shown in Fig. 4.

Using blocking layers, of different compositions in particular, it is not possible to indicate the most favorable substrate in terms of the highest absorbance, which would translate into the highest value of the generated photocurrent density. While the absorbance for all tested electrodes was very similar, it did not ensure uniform values for the photocurrents generated by the prepared devices.

### TiO_2_ and ZnO:TiO_2_ morphology

**Figure 5:**
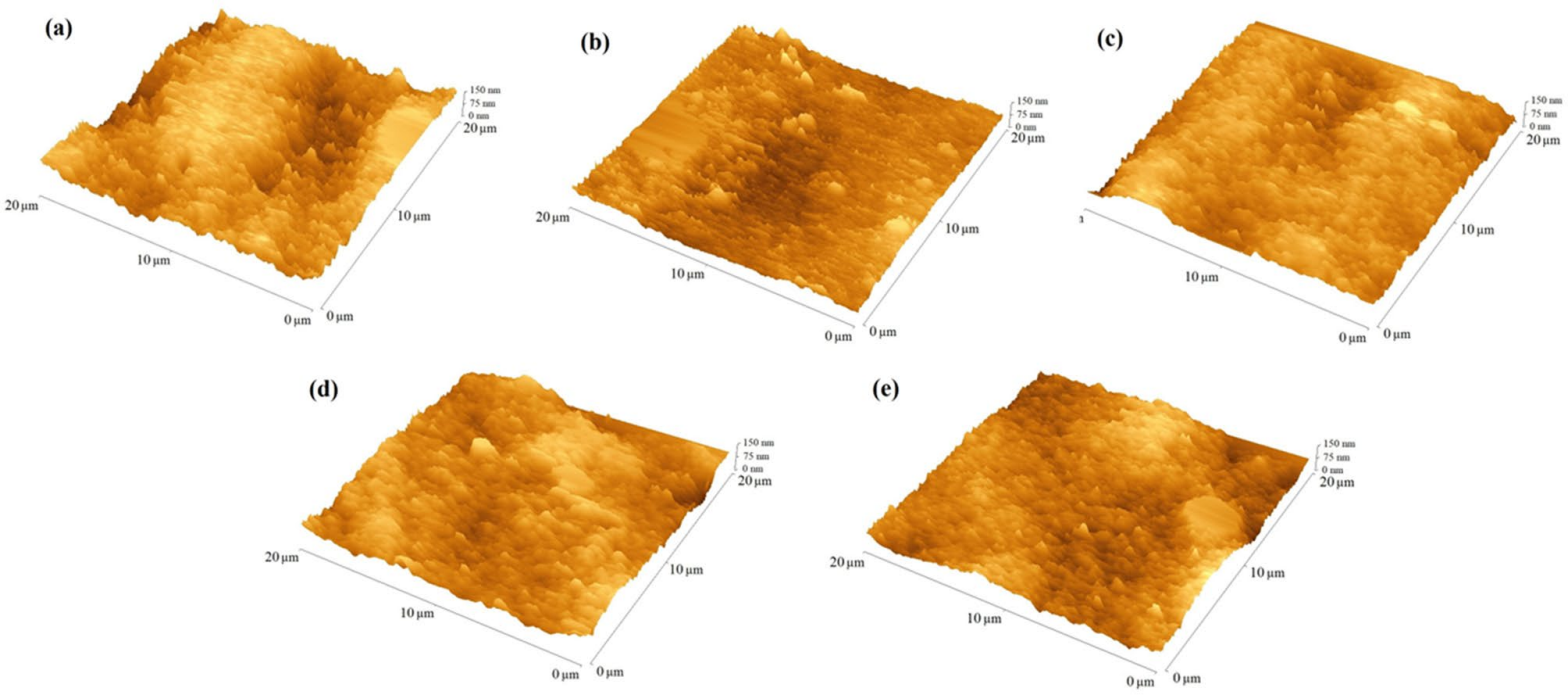
AFM micrographs of N719 absorber (**a**) sample ZnOTiO_2_, (**b**) sample Ti-Zn, (**c**) sample Ti-ZnO, (**d**) sample Ti, (**e**) reference sample without BL.

The surface quality of photoanodes was analyzed using atomic force microscopy (AFM) by determining the root-mean-square (RMS) which assess the roughness of the examined surface. The RMS values were similar for all the photoanodes—they were in the range of 40–45 nm. The R_a_ values were similar for all samples and were in the range of 33–39 nm. Notably, formations in the form of pyramids with a height of 115–128 nm were observed on the surface. It was found that the presence of the blocking layer did not affect the roughness of the TiO_2_ mesoporous layer with anchored dye. An example of AFM images of a surface is shown in Fig. 5. Obtained surface parameters are presented in Table 4.

**Table 4:** Surface parameters of the photoanodes studied.

Sample	R_a_ [nm]	RMS [nm]
ZnOTiO_2_	34	40
Ti-Zn	38	44
Ti-ZnO	39	45
Ti	33	40
Reference cell without BL	36	42

### Thickness and internal structure of blocking layers

**Figure 6:**
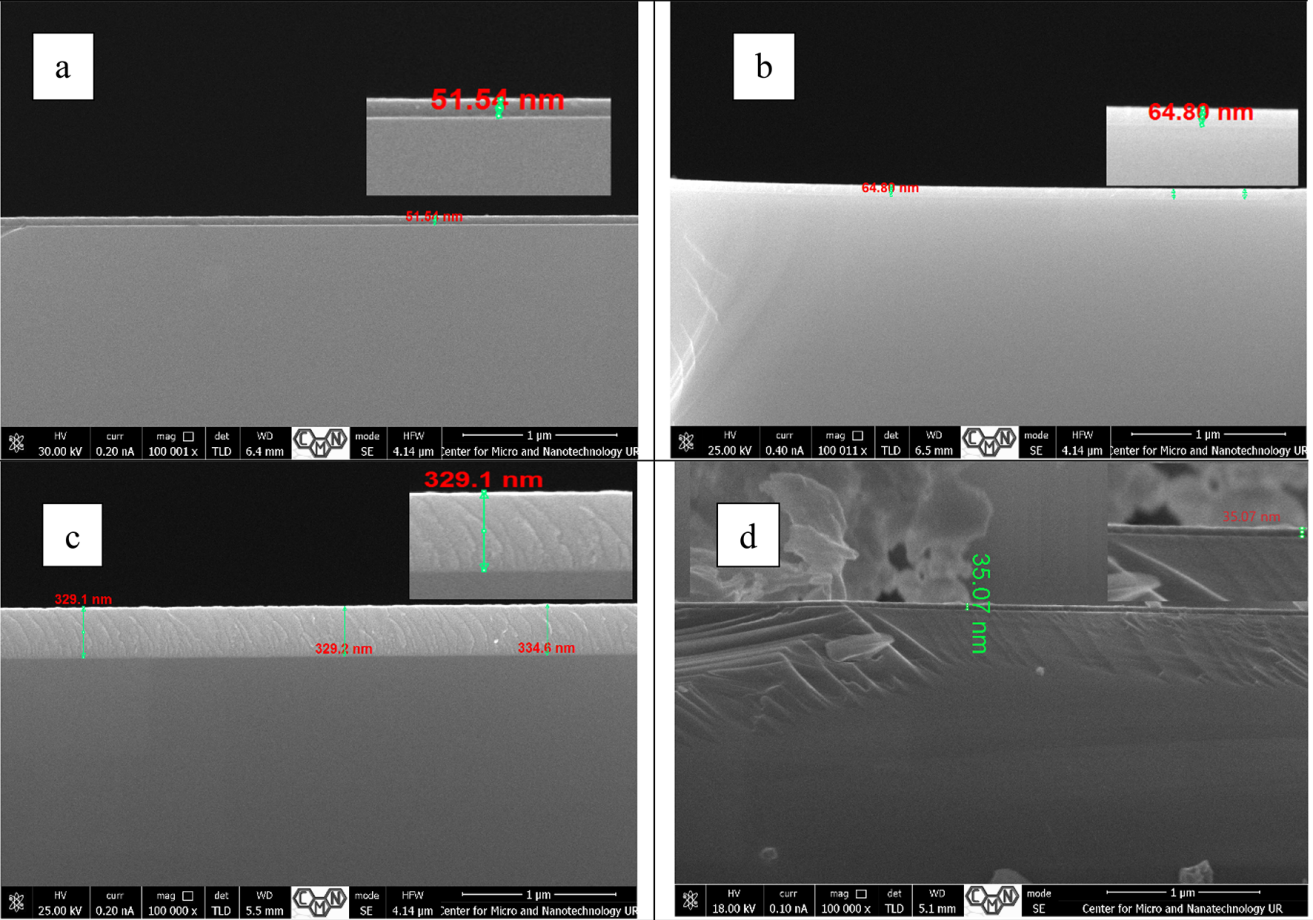
Blocking layer cross section for samples ZnOTiO_2_ (**a**), Ti-Zn (**b**), Ti-ZnO (**c**) and Ti (**d**).

Figure 6 presents cross sections of blocking layers produced for the parameters listed in Table 1. Layers used in this work as blocking layers in DSSC cells were selected as those that allowed the highest efficiencies to be obtained in TiO_2_:ZnO/Cu_x_O cells according to the previous group of experiments^41^.

For layer ZnOTiO_2_ with a thickness of 51 nm (Fig. 6a) and layer Ti with a thickness of 35 nm (Fig. 6d), we can see a clear interface between the substrate and the layer. For layer Ti-Zn (Fig. 6b) with a thickness of 67 nm and layer Ti-ZnO (Fig. 6c) with a thickness of 329 nm, we do not observe a breaking interface. Additionally, for layer Ti-ZnO, a homogeneous oriented structure with visible signs of columnar growth, was visible. Despite using a similar deposition time for layer Ti-ZnO (Fig. 6c) as for the other layers, it was significantly thicker, which may be associated with the accelerated growth rate, visible for the grains with clear column-grain structure. Both of these features impacted the final cell efficiency, which is visible in the I–V characterization.

### Obtained solar cells I–V characteristics

**Figure 7:**
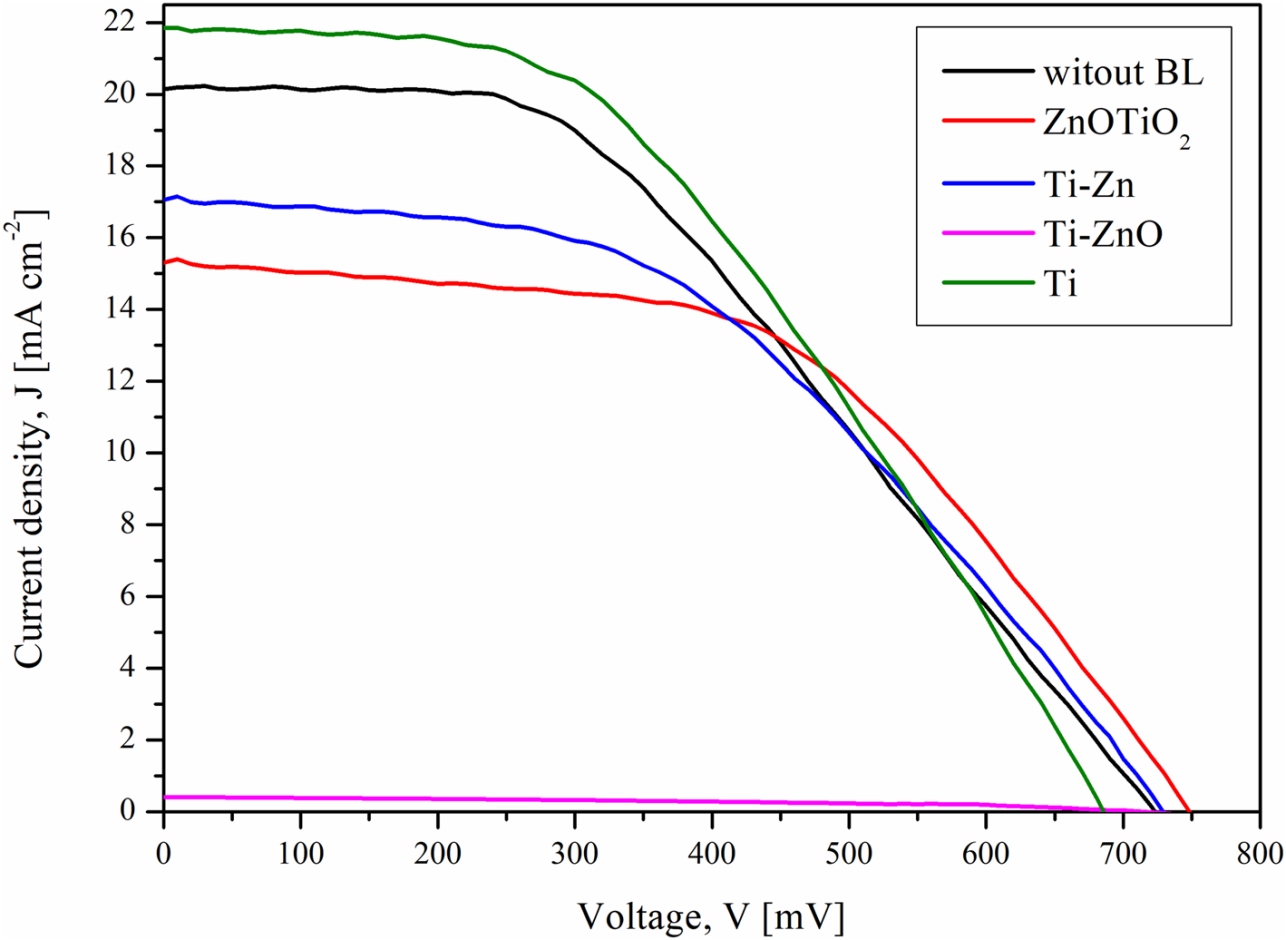
Photocurrent density–voltage characteristics of fabricated DSSCs.

The next step involved fabricating solar cells from previously studied photoanodes. The structure of prepared DSSCs devices was glass/FTO/BL/m-TiO_2_@N719/EL-HSE/Pt/FTO/glass. Based on the registered photocurrent density–voltage characteristics (Fig. 7), the basic photovoltaic parameters were determined: V_oc_—open circuit voltage, J_sc_—short circuit current density, FF and PCE are collected in Table 5.

**Table 5:** Photovoltaic parameters of prepared DSSCs.

Sample	Target material type and final blocking layer in brackets	V_oc_ [mV]	J_sc_ [mA cm^−2^]	FF	PCE [%]	Rs [Ohm]	Rsh [kOhm]
ZnOTiO_2_	ZnOTiO_2_, 4–3% at (TiO_2_ + ZnO)	749	15.31	0.52	6.09	35.162	2.895
Ti-Zn	Ti + Zn, centr 3.2 mm (TiO_2_ + ZnO)	730	17.06	0.46	5.73	36.520	3.273
Ti-ZnO	Ti + ZnO, centr 9.6 mm (TiO_2_ + ZnO)	715	0.41	0.42	0.12	629.950	12.298
Ti	Ti (TiO_2_)	686	21.84	0.44	6.59	29.753	1.636
Reference cell	No blocking layer	723	20.19	0.42	6.13	34.311	4.548

Based on the collected photovoltaic parameters, we were able to determine the influence of the blocking layer’s presence, its material composition, and the preparation conditions. Additionally, a reference solar cell without a blocking layer was prepared under identical conditions.

It was found that the use of a blocking layer containing only TiO_2_ nanoparticles, deposited with the help of commercially available titanium with (BL Ti) resulted in the highest efficiency. In this case, 7% increase in the performance of the DSSC device relative to the reference solar cell (6.29%), was achieved. The efficiency of the device thusly prepared was 6.74%. This result was mostly caused by the highest Rsh and lowest Rs values of all obtained layers.

In sample Ti-ZnO the addition of ZnO to TiO_2_ caused a decrease in device efficiency, mainly due to the decrease in the J_sc_ value in the range of 4.78–21.43 mA cm^−2^ in relation to a device containing BL Ti. Conversely, the V_oc_ values increased relative to the device containing the TiO_2_ blocking layer in the range of 29–63 mV. In most cases, the FF values remained at a similar level, except for the solar device with a photoanode containing a ZnO/TiO_2_ of 4–3% at ZnOTiO_2_ (TiO_2_ + ZnO) blocking layer, for which FF was 0.52.

Additionally, it was observed that the highest optical absorbance of the photoanode with BL sample Ti-ZnO did not correspond to high energy conversion efficiency. This sample’s extremely thick blocking layer reduced dramatically, and obtained cell photocurrent value through the rising of the series’ resistance. Even the preferred column-type grain growth of this layer could not compensate for this deteriorating effect. The inferior sample parameters may be possibly omitted after significant thickness reduction, which may be achieved in future through a radical process of time reduction. Analyzing Rs, it can be seen that the use of a TiO_2_ blocking layer allowed the lowest value of 29.753 Ohm to be reached. In addition, it should be taken into account, it was for this device that the highest value was obtained (21.84 mA cm^−2^).

## Conclusions

In the existing literature^42–44^, one can find publications indicating that the use of a ZnO blocking layer or mixture of ZnO and TiO_2_ results in an improvement in device efficiency, however, it is very important each time to do the selection of appropriate conditions for the preparation of blocking layers. This indicates that in this case it is possible to modify the methodology of preparation for the blocking layers, resulting in an increase in the efficiency of the devices. Here, in reported investigations the proper deposition technology of the blocking layer was verified, which resulted in a significant improvement of the photocurrent density and photoconversion efficiency. In this experiment, it was also proven that the admixture of ZnO phase inspires V_oc_ and FF growth, but is overall unfavorable according to the cell efficiency. Also, a smaller thickness of the blocking layer (below 100 nm) seems to be favorable for the final cell performance. The presented research is very important for further work aimed at optimising methods of preparing blocking layers containing both ZnO alone and mixtures of ZnO with TiO_2_. The best results (6.74%) were obtained for the device containing the blocking layer depositing TiO_2_ (Ti) while the lowest performance was characteristic of the solar cell containing BL Ti-ZnO (0.12%). Difference in thickness of the blocking layers (Ti-ZnO, 329 nm and Ti, 35 nm) leading to high resistance. According to the BL comparison one may clearly observe that the sample ZnOTiO_2_, 4–3% at (TiO_2_ + ZnO) is the most favourable solution with the final PCE value of 6.09%.
